# County-Scale Spatial Distribution of Soil Enzyme Activities and Enzyme Activity Indices in Agricultural Land: Implications for Soil Quality Assessment

**DOI:** 10.1155/2014/535768

**Published:** 2014-12-31

**Authors:** Xiangping Tan, Baoni Xie, Junxing Wang, Wenxiang He, Xudong Wang, Gehong Wei

**Affiliations:** ^1^College of Natural Resources and Environment, Northwest A&F University, Yangling, Shaanxi 712100, China; ^2^College of Life Science, Northwest A&F University, Yangling, Shaanxi 712100, China

## Abstract

Here the spatial distribution of soil enzymatic properties in agricultural land was evaluated on a county-wide (567 km^2^) scale in Changwu, Shaanxi Province, China. The spatial variations in activities of five hydrolytic enzymes were examined using geostatistical methods. The relationships between soil enzyme activities and other soil properties were evaluated using both an integrated total enzyme activity index (TEI) and the geometric mean of enzyme activities (GME). At the county scale, soil invertase, phosphatase, and catalase activities were moderately spatially correlated, whereas urease and dehydrogenase activities were weakly spatially correlated. Correlation analysis showed that both TEI and GME were better correlated with selected soil physicochemical properties than single enzyme activities. Multivariate regression analysis showed that soil OM content had the strongest positive effect while soil pH had a negative effect on the two enzyme activity indices. In addition, total phosphorous content had a positive effect on TEI and GME in orchard soils, whereas alkali-hydrolyzable nitrogen and available potassium contents, respectively, had negative and positive effects on these two enzyme indices in cropland soils. The results indicate that land use changes strongly affect soil enzyme activities in agricultural land, where TEI provides a sensitive biological indicator for soil quality.

## 1. Introduction

Soil is a natural resource playing key roles in organic matter (OM) decomposition, nutrient cycling, and water retention and release [1]. Soils are subject to natural or environmental degradation, often accompanied by erosion and leaching. Degradation of soils occurs even without the intervention of human agricultural practices [2, 3], thus threatening this valuable resource. Soil quality, particularly in arid and semiarid areas, needs to be preserved and improved for food security and environmental protection [4]. Previously, a variety of quantitative measures, including soil physicochemical properties indicative of the fundamental context of soil functions, have been extensively used to assess soil quality [5]. However, most soil physicochemical properties change slowly in response to the environmental stress, with significant changes commonly detected only after many years. By contrast, soil biological properties are sensitive indicators for soil quality, which rapidly respond to minor environmental changes in the soil [6].

Soil enzyme activity is a potential indicator of soil quality due to its high sensitivity to external interference and the ease of measurement [7]. The activities of hydrolytic enzymes are frequently measured to evaluate the effect of land use on biological processes in soils related to carbon (C), nitrogen (N), phosphate (P), and sulfur (S) cycling [8, 9]. Soil invertase deserves special recognition because its substrate, sucrose, is one of the most abundant soluble sugars in plants and is partially responsible for the breakdown of plant litter in soils [10]. Urease enzyme is responsible for the hydrolysis of urea fertilizer into NH_3_ and CO_2_ with a concomitant rise in soil pH [11]. Phosphatases are a broad group of enzymes that catalyze hydrolysis of esters and anhydrides of phosphoric acid. Apart from being a good indicator of soil fertility, phosphatase enzymes play key roles in the soil system [8]. Additionally, dehydrogenase enzyme activity is commonly used as an indicator of biological activity in soils [11]. Catalase activity in soils is considered to be an indicator of aerobic microbial activity and has been related to both the number of aerobic microorganisms and soil fertility [12].

Enzyme activity generally increases with the rise of soil organic matter (OM) content. Higher enzyme activity indicates larger microbial communities and greater stability of enzymes adsorbed on humic materials [13]. The activities of extracellular enzymes in soil vary significantly with seasons and geographical locations [14], as well as soil depth [15, 16]. Together these findings indicate that soil enzyme activities have broad-scale spatial variability depending on the environmental conditions. Due to seasonal and spatial variability, single biological properties cannot be accurate measures of soil quality [17, 18]. Therefore, multiparametric indices are recommended for environmental impact assessment of agroecosystems and nonagricultural soils [19, 20]. In fact, existing multiparametric indices have been found less sensitive to seasonal variations [21] than single properties.

Conventional statistical procedures assume that variations in soil properties are randomly distributed within sampling units. However, soil properties are continuous variables whose values at any location are expected to vary to different extents according to the direction and spacing of sampling points. Therefore, increasing emphasis has been put on the fact that variations in a soil property are not entirely random within a field. Such spatial structure of soil property should be taken into account in processing data [22]. Knowledge regarding the spatial distribution of soil enzyme activity across the landscape has great implications for interpreting the spatial pattern of OM decomposition and the rate of nutrient mineralization at regional scales [23]. When taking biological properties as the indicator of soil quality, it is necessary to consider the spatial variability of biological properties themselves as well as the underlying influencing factors [24]. Most studies have investigated the spatial variability of soil enzyme activities based on pot and/or microplot experiments [25, 26], while few reports are available at regional scales [22–24, 27–29]. Because the experimental data are not always applicable to actual field conditions, it is necessary to carry out field studies on the spatial variability of soil enzyme activity, especially in arid and semiarid areas associated with serious soil erosion.

The present study was conducted on China's Loess Plateau, which is known for its deep deposits of loess. Frequent and long-term anthropogenic activities have negatively affected the soil environment on the Loess Plateau, resulting in significant degradation of natural vegetation and intense soil erosion [30, 31]. Although a number of surveys have quantified soil erosion and the spatial variability of soil properties in the plateau region [32, 33], there is little information on the spatial distribution of soil enzyme activities across the large-scale landscape. The responses of soil enzyme activities to geographical locations on the Loess Plateau remain unclear, and related research is urged to provide reference data for integrated soil quality management.

The objectives of this study were (1) to quantify the activities of five soil enzymes (invertase, urease, phosphatase, catalase, and dehydrogenase) and selected soil physicochemical properties across an entire county (Changwu) on the Loess Plateau; (2) to investigate the spatial variability of soil enzyme activities in a representative area in the Hilly-Gully Region of Loess Plateau; and (3) to explore the relationships between soil enzyme activities and physicochemical properties using an integrated soil enzyme activity index (TEI) and to compare it with the geometric mean of enzyme activities (GME).

## 2. Materials and Methods

### 2.1. Study Area

**Figure 1:**
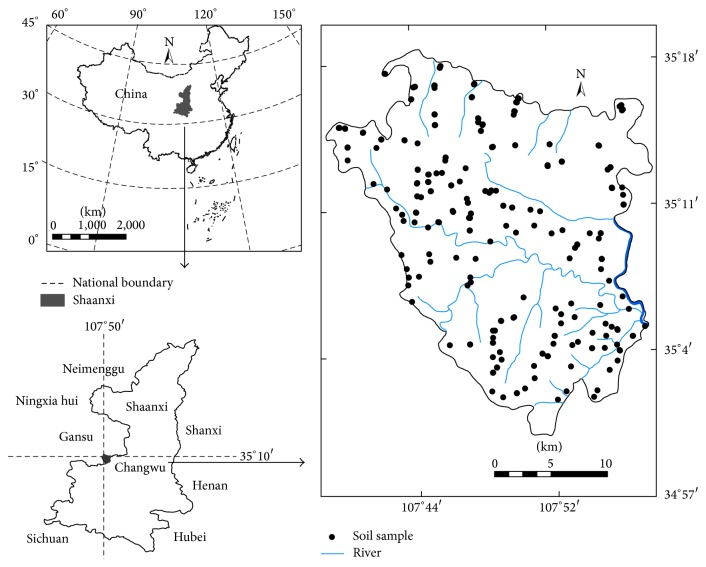
Location of Changwu County and distribution of sampling points in the study area.

Changwu County (567 km^2^) is located in Xianyang City, Shaanxi Province, China (34°59′–35°8′N, 107°17′–107°58′E) (Figure 1). This county is part of the Hilly-Gully Region of Loess Plateau. It mainly consists of low, rolling hills and deep, narrow gullies. The dominant soil types are Cumuli-Ustic Isohumosols (dark loessial soil) and Loessi-Orthic Primosols (cultivated loessial soils). The altitude ranges from 847 to 1274 m.a.s.l. Changwu has a continental semiarid monsoon climate, with mean monthly temperatures ranging from −9.9°C in January to 24.4°C in July. The annual average temperature is 9.2°C, and the annual precipitation is 573 mm. Heavy rainstorms occasionally occur in this county, mainly between June and September. The driest season is during winter, from December to February.

### 2.2. Soil Sampling and Chemical Analysis

In late October 2008, soils were sampled at the 0–20 cm depth from 245 locations in 171 villages of Changwu (Figure 1). The samples from croplands were taken randomly in every village and those from apple orchards were randomly taken in every two villages across the county. The sampling locations were identified using a global positioning system (GARMIN GPS72). One soil sample in the cropland consisted of five individual subsamples which were taken randomly within a 10 m radius from each sampling point. Similarly, for a sample in apple orchard, five subsamples were taken from each of three rows. There were 170 soil samples from croplands and 75 from apple orchards. All soil samples were air-dried at room temperature and then passed through a 1.0 mm sieve.

Soil physicochemical analysis was conducted on 0.25 mm sieved samples using routine analytical methods [34]. The OM content was determined by oxidation with K_2_Cr_2_O_7_/H_2_SO_4_. Total N content was analyzed following the Kjeldahl digestion procedure. Alkali-hydrolyzable N content was measured using the alkaline hydrolysis diffusion method. For total P and K analyses, the samples were decomposed with sodium hydroxide (solid) at 720°C and extracted with hot water, followed by molybdenum blue spectrophotometry and atomic absorption spectrophotometry, respectively. Available P was extracted with 0.5 mol·L^−1^ sodium bicarbonate and quantified by molybdenum antimony blue spectrophotometry. Available K was extracted with 1 mol·L^−1^ ammonium acetate and quantified by atomic absorption spectrophotometry. Soil particle size distribution was determined using a pipette method. Cation exchange capacity (CEC) was quantified using a 1 mol·L^−1^ ammonium acetate exchange method. Soil pH was measured in a 5 : 1 water  to soil slurry with an electronic pH meter (Mettler Toledo FE20).

### 2.3. Soil Enzyme Activity Assays

Enzyme activities were measured with 1 mm sieved soil samples in unbuffered extract solutions meant to simulate the field conditions.

Invertase activity was determined as described by [35]. Briefly, 5 g of air-dried soil was mixed together with 15 mL of 8% sucrose solution, 5 mL of distilled water, and 5 drops of toluene. After incubation for 24 h, at 37°C, the soil solution was centrifuged at 4000 rpm for 5 min and a 1 mL aliquot was transferred to a volumetric flask containing 3 mL of 3,5-dinitrosalicylic acid. The mixture was heated for 5 min. When the solution reached room temperature, glucose content was quantified colorimetrically at 508 nm on a spectrophotometer (INESA 722N). Invertase activity was expressed as *μ*g glucose·g^−1^ soil·h^−1^.

For the urease activity assay, 5 g of air-dried soil was mixed with 5 mL of toluene, 20 mL of distilled water, and 10 mL of 10% urea solution. After incubation at 37°C for 24 h, the soil suspension was centrifuged at 4000 rpm for 5 min and a 1 mL aliquot was treated with 4 mL of sodium phenol solution (containing 100 mL of 6.6 M phenol solution and 100 mL of 6.8 M NaOH) and 3 mL of 0.9% sodium hypochlorite solution. The ammonium released into the solution was quantified colorimetrically at 578 nm on a spectrophotometer. Urease activity was expressed as *μ*g NH_4_-N·g^−1^ soil·h^−1^ [35].

For phosphatase activity assay, 5 g of air-dried soil was mixed with 5 drops of toluene, 10 mL of disodium phenyl phosphate solution, and 10 mL of distilled water. The suspension was incubated for 24 h, at 37°C, and then centrifuged at 4000 rpm for 5 min. The supernatant was colored with 0.25 ammonia-ammonium chloride buffer, at pH 9.6, 0.5 mL of 2% 4-aminoantipyrine, and 0.5 mL of 8% potassium ferrocyanide. The phenol content was determined colorimetrically at 510 nm on a spectrophotometer. Phosphatase activity was expressed as *μ*g phenol·g^−1^ soil·h^−1^ [35].

Catalase and dehydrogenase activities were assayed using the method of [11]. For catalase activity assay, 2 g of air-dried soil was mixed with 40 mL of distilled water and 5 mL of 0.3% H_2_O_2_. The soil slurry was shaken for 20 min at 150 rpm. The remaining peroxide was stabilized by adding 5 mL of 1.5 M sulfuric acid and the solution was then immediately centrifuged at 4000 rpm for 5 min. The peroxide in the supernatant was titrated with 0.05 M KMnO_4_. Catalase activity was expressed as mL KMnO_4_·g^−1^ soil·h^−1^.

For dehydrogenase activity assay, 3 g of air-dried soil was mixed with a 3% triphenyl tetrazolium chloride solution as the substrate and 1.25 to 1.75 mL of distilled water. The soil slurry was mixed thoroughly and incubated at 37°C for 24 h. Thereafter, triphenyl formazan was extracted with methanol and quantified by colorimetric analysis at 485 nm. Dehydrogenase activity was expressed as *μ*g TPF·g^−1^ soil·h^−1^ [11] and all enzyme activities were calculated as the mean of two replicates.

### 2.4. Statistical and Geostatistical Analyses

Descriptive statistics (arithmetic mean, maximum and minimum, median, standard deviation, coefficient of variation, skewness, and kurtosis), and Pearson product moment correlation analysis were conducted using SPSS 18 for Windows (SPSS Inc., Chicago, IL, USA). Data normality was tested by one-sample Kolmogorov-Smirnov test.

The semivariogram analysis was used to assess the spatial structure of the studied variables. Then, the Ordinary Kriging interpolation was used to estimate the unknown values at unsampled locations and to map the spatial variability of soil properties and TEI [36]. The sample semivariogram was calculated using

(1) $$\begin{array}{c} \gamma \left(h\right)=\frac{1}{2N\left(h\right)}\overset{N\left(h\right)}{\underset{i=1}{\sum }}{\left[Z\left(x_{i}\right)-Z\left(x_{i}+h\right)\right]}^{2}, \end{array}$$

where *γ*(*h*) is the semivariance for interval distance class *h*, that is, the distance separating sample points *x*_*i*_ and *x*_*i*_ + *h*, *N*(*h*) is the number of sample couples for the lag interval *h*, and *Z*(*x*_*i*_) and *Z*(*x*_*i*_ + *h*) are measured values at points *i* and *i* + *h*, respectively.

Three variogram models (spherical, Gaussian, and exponential) were fitted to the sample semivariograms in this research. The best fitted model should have the smallest residual sum of squares (RSS) and the largest coefficient of determination (*R* ^2^) between predicted values and the measured values of soil properties. Then the best fitted models were used to provide input parameters for Kriging interpolation. The estimated values were obtained using

(2) $$\begin{array}{c} Z^{*}\left(x_{0}\right)=\overset{n}{\underset{i=1}{\sum }}\lambda_{i}Z\left(x_{i}\right), \end{array}$$

where *Z* ^*^(*x*_0_) is the predicted value at point *x*_0_, *Z*(*x*_*i*_) are the measured values at sampling location *x*_*i*_, *λ*_*i*_ is the weight to be assigned to sample *x*_*i*_, and *n* is the number of sites within the neighborhood searched for the interpolation.

Here log or Box-Cox transformation was used when the original data was not normally distributed. Semivariance calculations of the soil properties were conducted based on the maximum sampling distance of 17 km, which was divided into 15 lag distance classes separated by an average of 1.1 km. No significant anisotropy was considered, because the anisotropy ratio was less than 2.5 [37]. The cross validation procedure was used to assess the models fitted to experimental semivariograms. After a semivariogram model has been obtained, the Kriging technique was applied to obtain a map of estimates. The geostatistical analysis was performed in ArcGIS (version 10.0, ERIS, Redlands, CA, USA).

### 2.5. Calculation of Soil Enzyme Activity Indices

The integrated total enzyme activity index (TEI) was calculated using the following equation [20]:

(3) $$\begin{array}{c} \text{TEI}=\overset{i}{\underset{n=1}{\sum }}\frac{X_{i}}{\bar{X_{i}}}\;\left(n=1,2,3,4,5\right), \end{array}$$

where *X*_*i*_ is the activity of soil enzyme *i* and $\bar{X_{i}}$ is the mean activity of enzyme *i* in all samples.

The geometric mean of enzyme activities (GME) was calculated by (4) discussed elsewhere [38] as

(4) GME=Invertase×Urease×Phosphatase×Catalase×Dehydrogenase5.

## 3. Results

### 3.1. Descriptive Statistics of Soil Properties

The OM content of surface soil samples averaged 12.57 g·kg^−1^ and the total N concentration averaged 0.89 g·kg^−1^ across the county. Both parameters varied substantially, from 5.16 to 18.25 g·kg^−1^ for OM content and from 0.28 to 1.37 g·kg^−1^ for total N content. The soils were mostly fine in texture, with an average clay content of 33%. Soil pH ranged from 7.80 to 9.09, with a mean of 8.59 (Table 1).

Invertase activity of surface soil samples (0–20 cm depth) ranged from 102 to 707 *μ*g glucose·g^−1^ h^−1^, with a mean of 379 *μ*g glucose·g^−1^ h^−1^. Urease activity ranged from 3.16 to 108 *μ*g NH_4_^+^-N·g^−1^ h^−1^, with a mean of 25.0 *μ*g NH_4_^+^-N·g^−1^ h^−1^. Phosphatase activity ranged from 15.1 to 71.6 *μ*g phenol·g^−1^ soil·h^−1^, with a mean of 34.88 *μ*g phenol·g^−1^ soil·h^−1^. Catalase activity ranged from 4.58 to 13.1 mL KMnO_4_·g^−1^ soil·h^−1^, with a mean of 8.79 mL KMnO_4_·g^−1^ soil·h^−1^. Dehydrogenase activity ranged from 0.15 to 3.06 *μ*g TPF·g^−1^ h^−1^, with a mean of 25.0 *μ*g TPF·g^−1^ h^−1^ (Table 1). The coefficients of variation (CV) were 28% for invertase activity, 53% for urease activity, 25% for phosphatase activity, 22% for catalase activity, and 49% for dehydrogenase activity.

The activities of all enzymes and the levels of OM, AN, AK, and CEC were normally distributed (one-sample Kolmogorov-Smirnov test, *P* > 0.05). Total N and pH levels were negatively skewed and nonnormally distributed, whereas total P, total K, available P, and clay contents were positively skewed and nonnormally distributed. The underlying reasons for normal or nonnormal distribution of these variables were unknown, but management and spatial effects seemed to play a role.

### 3.2. Relationships of Soil Physicochemical Properties and Enzymatic Activities

Results of the correlation analysis in all soil samples showed that the OM content was significantly correlated with invertase, urease, phosphatase, and dehydrogenase activities (*P* < 0.01) but not with catalase activity (*P* > 0.05). The alkali-hydrolyzable N content was strongly correlated with invertase, urease, phosphatase, catalase, and dehydrogenase activities (*P* < 0.01). No significant correlation was found between soil pH and phosphatase activity (*P* > 0.05). However, soil pH was positively correlated with dehydrogenase activity (*P* < 0.05) and negatively correlated with invertase, urease, and catalase activities (*P* < 0.01; Table 2). Additionally, soil phosphatase activity was extremely significantly correlated with invertase (*r* = 0.409, *P* < 0.01) and urease activities (*r* = 0.228, *P* < 0.01), and catalase activity was significantly correlated with invertase (*r* = 0.146, *P* < 0.05) and urease activities (*r* = 0.144, *P* < 0.05). Soil dehydrogenase activity was significantly correlated with invertase (*r* = 0.519, *P* < 0.01), phosphatase (*r* = 0.649, *P* < 0.01), and catalase activities (*r* = −0.174, *P* < 0.01).

### 3.3. Spatial Structure of Soil Properties

Semivariance analysis showed that the soil properties generally had spatial dependence (Table 3). The semivariograms all exhibited spatial structure. The experimental semivariograms for soil dehydrogenase activity, available K content, CEC level, and clay content were fitted by exponential models. The experimental semivariogram for total K content was fitted by a spherical model. The experimental semivariograms for other soil properties were fitted by the Gaussian models.

The spatial dependence of the data was confirmed by total variance (Sill) composed of structural (C) and nugget variances (Co). Nugget to sill ratio ([Co/Sill]) for soil enzyme activities was 67% and that for soil physicochemical properties was 54%. Nugget to sill ratios of urease and dehydrogenase activities accounted for 85% and 71% of the total variance, respectively. These values were significantly higher than sill and nugget effects. Available K content had the largest nugget to sill ratio among all soil properties. The effective ranges of phosphatase and urease activities were greater than those of invertase, catalase, and dehydrogenase activities (3.9, 5.3, and 2 km, resp.).

**Figure 2:**
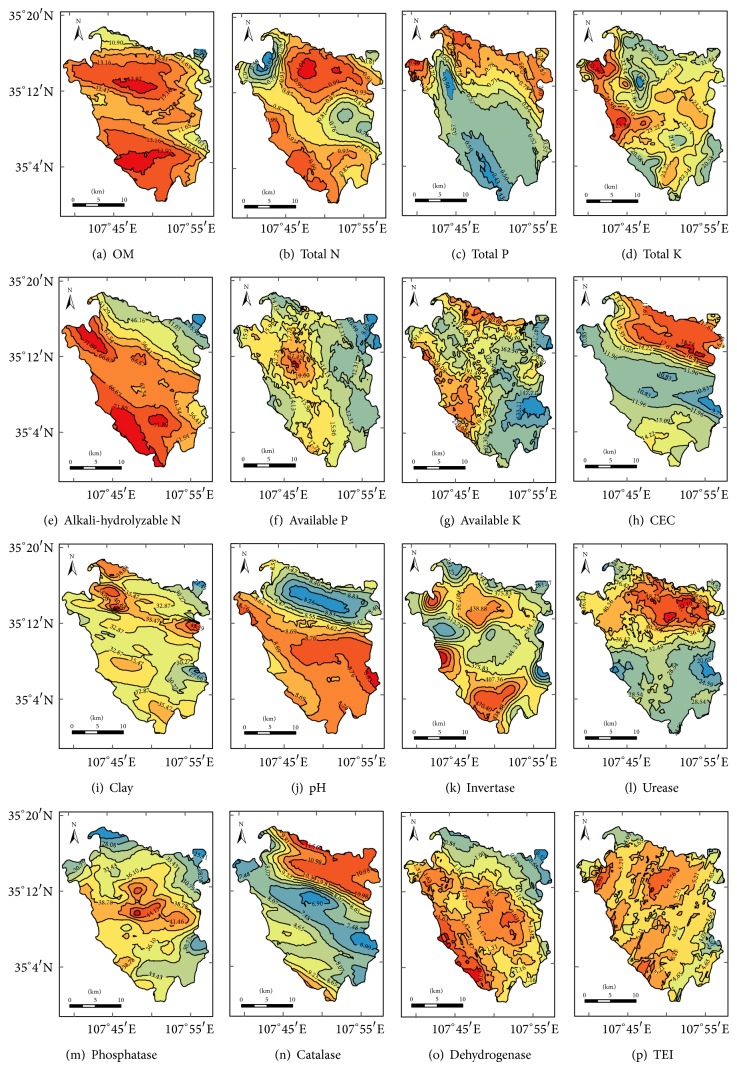
Spatial distribution patterns of soil physicochemical properties and enzyme activities and TEI in surface horizon of Changwu County.

The Kriging maps showed that soil OM, total N, and CEC levels showed similar spatial distribution patterns, with the lowest values occurring in the center of the county (Figures 2(a), 2(b), and 2(h)). Soil alkali-hydrolyzable N, available P contents, and pH value were highest in the central and southern parts and lowest in the northern part of Changwu (Figures 2(e), 2(f), and 2(j)). In contrast, total P content increased from the south to the northwest (Figure 2(c)). Soil total K, available K, and clay contents had no obvious variation trends across the county (Figures 2(d), 2(g), and 2(i)). The distribution of these three properties did not correspond to the topographical feature of the study area or to the spatial distribution of the other soil properties. Soil invertase, urease, and catalase activities were highest in the northern area in Changwu County, followed by the central and southern areas (Figures 2(k), 2(l), and 2(n)). Soil phosphatase activity was highest in the center of the county (Figure 2(m)). Dehydrogenase activity decreased from the southwest to the northeast (Figure 2(o)).

### 3.4. Enzymatic Activity Indices

A main novelty of this study was to introduce the integrated index TEI as a dimensionless parameter for easy comparison of the combined enzyme activity and the quality of each soil sample. We also compared this index with the commonly used GME index. The TEI values of total, orchard, and cropland soil samples varied from 1.87 to 7.43, 2.7 to 7.4, and 1.8 to 7.4, with a median value of 5.07, 4.9, and 5.07, respectively. The mean TEI value of all soil samples was estimated to be 5. The GME values of total, orchard, and cropland soil samples varied from 6.9 to 33, 10 to 30, and 22 to 33, with a median value of 21.6, 20.8 and 22.1, respectively. The mean GME values of different groups of soil samples were estimated to be 21.3, 20.5, and 21.7, respectively.

The TEI and GME values were most correlated with the activities of invertase, urease, phosphatase, catalase, and dehydrogenase, except orchard soil catalase activity (Table 4). Pearson correlation analysis showed that TEI and GME were positively correlated with soil OM, total N, and alkali-hydrolyzable N contents but negatively correlated with pH level. In addition, the TEI and GME values of total and orchard soil samples were positively correlated with total K content. The TEI and GME values of total and cropland soil samples were positively correlated with available P and K contents. The TEI values of total and cropland soil samples were positively correlated with CEC (Table 4).

Multivariate regression analysis was carried out to investigate the relationship between soil physicochemical properties and enzyme activity indices (TEI and GME) (Table 5). Among the soil properties measured in this equation, soil OM content had the strongest positive effect while soil pH had a negative effect on the two indices. Additionally, total P content had a positive effect on TEI and GME in orchard soils. The alkali-hydrolyzable N and available K contents had negative and positive effects on TEI and GME in cropland soils while the alkali-hydrolyzable N and available P contents had positive and negative effects on both enzyme activity indices in total soils.

## 4. Discussion

### 4.1. Spatial Structure of Soil Enzyme Activities

Wilding [39] previously described a classification scheme for identifying the variability of soil properties based on their CV values. According to Wilding's classification, the CV values of soil invertase, phosphatase, and catalase activities are relatively small (22–28%), whereas those of urease and dehydrogenase activities (49–53%) are at the medium level. Soil enzyme activities have close relationships with soil biology and are sensitive in discriminating among soil management practices, such as fertilization by means of animal manure or green manures/crop residues and municipal refuse amendment, as well as among tillage treatments. However, a previous report indicated that dehydrogenase and cellulose activities in the surface soil horizon of an arable land (2 km^2^) had small CV values (18% and 26%, resp.) [26]. Bonmati et al. [25] observed that the CV values for soil urease, phosphatase, and protease activities ranged from 28% to 60% in a 15 m × 40 m meadow. Smith and Halvorson [24] reported the CV values of 33% for phosphatase activity and 36% for dehydrogenase activity in agricultural land (*n* = 220). These findings confirmed that the spatial variability of soil enzyme activities varied in different ecosystems.

Knowledge regarding the spatial variability of soil biological properties is critical for the management and improvement of agricultural soil quality. Reliable information on the range of spatial relationships makes it possible to define the sampling strategy needed for accurately mapping soil biological properties [22]. In the present study, the best model of semivariogram varied depending on the kinds of soil enzymes, and the effective ranges of soil enzymes decreased in the order of phosphatase→urease→catalase→invertase→dehydrogenase (Table 3). Semivariograms for enzyme activities exhibited spatial correlated distances that ranged from 2 to 13 km (Table 3). Likewise, Aşkın and Kızılkaya [22] recently had reported that the zone (4500 × 4500 m) of influence of soil urease activity was approximately 19.3 km. In general, however, field-scale (50 ha) studies indicate that the effective ranges of soil dehydrogenase and cellulose activities are 84.3–93.3 m [26]. The difference among those studies may depend highly on the size of sample area and the sampling distance. Both soil properties and scale effect have strong effects on the spatial distribution of enzyme activity. The spatial structure of soil properties is complex and contrasting range values have been reported. Therefore, future research should consider the size of study area and the distances between sampling points. The most appropriate sampling scheme and the separation distance between sampling points for future data collection are highly important and should be determined in preliminary studies [23].

The ratio of nugget to sill can be used as a criterion to classify the strength of the spatial dependence of soil properties (<25%, strong spatial dependence; 25–75%, moderate spatial dependence; and >75%, weak spatial dependence) [40]. In the present study, the nugget to sill ratios descended in the order of urease activity (85%) > dehydrogenase activity (71%) > phosphatase activity (61%) > invertase activity (60%) > catalase activity (54%). The activities of invertase, phosphatase, dehydrogenase, and catalase are more spatially dependent than urease activity. The moderate spatial dependence of invertase, phosphatase, dehydrogenase, and catalase activities indicates that these enzyme activities are primarily controlled by specific geological factors and have better correlation. The weak spatial dependence of soil urease activities indicates that the environment has a stronger impact than geographical distance on the spatial distribution of relevant microbial communities. Similarly, high nugget effects have been observed for dehydrogenase activity in no-till field which was cropped with corn (*Zea mays* L.) and soybean (*Glycine max* (L.) Merr.) (62.7%) [40], Cambisol soil under winter wheat (68.4%) [26], and no-till wheat crop soil (70.7%) [41]. The variability of weakly spatially dependent parameters may be caused by agricultural practices, such as application of fertilizers and tillage, whereas strongly spatially dependent parameters are influenced by variations in innate soil characteristics such as texture and mineralogy [40]. The differences in spatial dependency of soil enzyme activities may be related to different spatial distribution of various microbial groups and soil fertilizer, which reflects the influence of different soil topography, vegetation, and agricultural practices.

Visualizing the spatial distribution of soil biotic components contributes to the understanding of spatial structure and thus may help with accurate prediction and mapping of soil microbial properties [42]. The activities of five soil enzymes in Changwu County showed patchy distribution related to soil physicochemical properties tested (Figure 2). For example, invertase, urease, and catalase activities were generally highest in the northern part of Changwu. As for soil properties, the OM, total N, total P, and CEC levels were high while the alkali-hydrolyzable N, available P, available K, and soil pH levels were relatively low in the northern part of Changwu. Spatially different distribution of the enzymatic activity is related to the variations in soil OM content, the activity of related living organisms, and the intensity of biological processes [22]. Therefore, it is not surprising that microbial properties show cross-dependence among themselves and with other soil properties depending on the ecosystem [42]. Understanding of the spatial distribution of physicochemical indicators for soil quality is an important step for explaining the spatial variability of biological parameters. The statistical results analysis showed that there were highly significant correlations between soil enzyme activities (invertase, urease, phosphatase, catalase, and dehydrogenase) and several physicochemical properties (OM, total N, total P, alkali-hydrolyzable N, CEC, and pH levels) in Changwu. These strong relationships confirm that soil enzyme activities provide a meaningful integrative measure of soil physicochemical properties and biological soil fertility, which thus may play a role in monitoring soil biological quality [43].

### 4.2. Implications for Assessing Soil Quality by a Biological or an Integrated Enzyme Activity Index

Results of the multivariate regression analysis indicated that soil OM and pH levels, respectively, had stronger positive and negative effects than other soil properties on TEI and GME. Meanwhile, TEI and GME had stronger positive correlation with soil physicochemical properties than individual enzyme activities (Table 4). Generally, the OM content positively affects extracellular enzyme activity in soil [42, 43]. The adsorption of enzyme molecules to soil particles and OM materials possibly protected the enzymes against soil pH changes. Alternatively, this could be attributed to the dependence of microbial activity (hence enzyme production) on the supply of organic substrate (organic C availability). In such cases, the organic C content and enzyme activity would be related to each other via microbial biomass [26].

Soil biological properties are highly sensitive to environmental stress and thus can be used to assess quality. Any soil quality index should include several biological variables so as to better reflect the complex processes affecting soil quality and to compensate for the wide variations occurring in individual properties [44]. For instance, the TEI values showed more similar spatial distribution patterns with soil physicochemical properties (Figure 2(p), *n* = 245). As the TEI index introduced in this study involves only five soil enzymes, further large-scale studies are recommended to verify the applicability of TEI as an integrated activity index of soil enzymes in different ecosystems. A more comprehensive and accurate biological indicator for changes in soil quality can be obtained by taking into consideration more enzyme components and/or microbial parameters that are indicative of key soil biological processes. These indicators must be quantified on a local and landscape basis as a means for making small-scale and regional management decisions [24]. In view of the limitations of single soil biological properties, it is recommended to develop and use multiparametric indices such as TEI for providing and integrating more information on soil quality.

## 5. Conclusion

Results of the conventional and geostatistical analyses indicate that the spatial variability of agricultural soil properties is complex in Changwu County. Spatial distribution maps show that soil invertase, urease, and catalase activities are high in the northern area, soil phosphatase activity is high in the central area, and soil dehydrogenase activity is high in the southwestern area of the county. The spatial patterns of soil quality are better reflected using an integrated soil enzyme index, which provides a sensitive biological indicator for soil quality as compared with the single enzyme activities.

## Figures and Tables

**Table 1:** Descriptive statistics of selected soil physicochemical properties and enzyme activities in surface horizon (0–20 cm) of Changwu County, Shaanxi Province, China (*n* = 245).

Parameters	Range	Minimum	Maximum	Mean	Standard deviation	K-S Z Asymp. Sig. (2-tailed)		Skewness	Kurtosis
OM/g kg^−1^	13.09	5.16	18.25	12.57	2.22	1.21	0.11	−0.52	1.4
Total N/g kg^−1^	1.09	0.28	1.37	0.89	0.19	2.64	0.00	−0.46	0.78
Total P/g kg^−1^	1.45	0.17	1.62	0.67	0.26	2.51	0.00	1.14	1.95
Total K/g kg^−1^	11.42	16.68	28.1	22.23	1.95	2.23	0.00	0.19	0.1
Alkali-hydrolyzable N/mg kg^−1^	85.75	19.25	105	59.68	15.72	1.01	0.26	0.02	−0.04
Available P/mg kg^−1^	46.71	2.54	49.25	17.06	10.51	1.66	0.01	1.53	1.88
Available K/mg kg^−1^	450	63.13	513.13	199.36	94.22	0.76	0.62	0.8	−0.09
CEC/cmol kg^−1^	16.84	6.68	23.52	13.91	3.29	1.08	0.19	0.73	−0.12
Clay/%	33.09	17.41	50.5	33.19	5.14	1.59	0.01	0.51	2.27
pH	1.20	7.89	9.09	8.59	0.23	1.81	0.00	−0.64	0.14
Invertase/*μ*g glucose·g^−1^ soil·h^−1^	605.1	102.07	707.18	379.26	106.34	0.79	0.55	0.32	0.04
Urease/*μ*g NH_4_-N g^−1^ soil·h^−1^	104.7	3.16	107.86	37.86	20.07	1.38	0.05	0.77	0.3
Phosphatase/μg phenol·g^−1^ soil·h^−1^	56.55	15.12	71.67	34.88	8.83	0.79	0.57	0.57	1.24
Catalase/mL KMnO_4_·g^−1^ soil·h^−1^	8.55	4.58	13.13	8.79	1.97	0.86	0.44	0.11	−0.64
Dehydrogenase/*μ*g TPF·g^−1^ soil·h^−1^	2.91	0.15	3.06	1.29	0.63	0.99	0.28	0.48	−0.28

K-S Z, Kolmogorov-Smirnov Z; OM, organic matter; N, nitrogen; P, phosphorous; K, potassium; and CEC, cation exchange capacity.

**Table 2:** Correlation coefficients (Pearson *r* value) between soil physicochemical properties and enzyme activities in surface horizon (0–20 cm) of Changwu County (*n* = 245).

	Invertase	Urease	Phosphatase	Catalase	Dehydrogenase
OM	0.547^**^	0.386^**^	0.580^**^	−0.06	0.469^**^
Total N	0.300^**^	0.431^**^	0.243^**^	0.255^**^	0.03
Total P	−0.06	0.317^**^	−0.176^**^	0.315^**^	−0.325^**^
Total K	0.11	0.11	0.12	−0.213^**^	0.246^**^
Alkali-hydrolyzable N	0.393^**^	0.192^**^	0.486^**^	−0.462^**^	0.552^**^
Available P	0.05	0.366^**^	0.09	−0.186^**^	−0.02
Available K	0.00	0.358^**^	0.147^*^	0.09	−0.06
CEC	0.11	0.291^**^	−0.13	0.718^**^	−0.322^**^
Clay	0.11	0.127^*^	0.02	0.297^**^	−0.10
pH	−0.167^**^	−0.380^**^	0.05	−0.529^**^	0.154^*^

∗ and ∗∗ represent statistical significances at the 5% and 1% levels, respectively; OM, organic matter; N, nitrogen; P, phosphorous; K, potassium; and CEC, cation exchange capacity.

**Table 3:** Parameters for variogram models of soil physicochemical properties, enzyme activities, and TEI in surface horizon (0–20 cm) of Changwu County (*n* = 245).

Parameters	Model	*C*_0_	*C*_0_ + *C*	[*C*_0_/(*C*_0_ + *C*)]100	Range/km	RMSS
OM	Gaussian	3.03	4.36	69.53	9.3	1.09
Total N	Gaussian	0.02	0.04	57.05	7.49	1.05
Total P	Gaussian	0.04	0.05	77.74	6.26	1.05
Total K	Spherical	0.89	3.07	28.83	3.31	1.01
Alkali-hydrolyzable N	Gaussian	100.93	243.7	41.41	11.33	1.06
Available P	Gaussian	0.26	0.32	81.78	15.63	1.08
Available K	Exponential	0.19	0.21	89.89	10.27	1.01
CEC	Exponential	0.01	0.04	31.06	10.46	1.01
Clay	Exponential	7.18	24.64	29.14	5.23	0.97
pH	Gaussian	0.02	0.05	37.78	9.36	1.03
Invertase	Gaussian	16.89	27.93	60.45	3.89	1.00
Urease	Gaussian	8.87	10.4	85.34	9.36	1.02
Phosphatase	Gaussian	1.59	2.6	61.31	12.99	1.01
Catalase	Gaussian	1.32	2.44	54.27	5.33	0.98
Dehydrogenase	Exponential	0.19	0.27	71.26	1.99	0.96
TEI	Gaussian	0.65	1.30	50.15	0.84	1.07

*C*_0_, nugget variance; *C*, structural variance; *C*_0_ + *C*, sill; RMSS, root-mean-square standardized; OM, organic matter; N, nitrogen; P, phosphorous; K, potassium; and CEC, cation exchange capacity.

**Table 4:** Pearson correlation coefficients between soil physicochemical properties and enzyme activity indices in surface horizon (0–20 cm) of Changwu County.

	Samples					
	Orchard		Cropland		Total	
	(*n* = 75)		(*n* = 170)		(*n* = 245)	
Enzyme activity index	TEI	GME	TEI	GME	TEI	GME
OM	0.566^**^	0.569^**^	0.696^**^	0.693^**^	0.659^**^	0.655^**^
Total N	0.299^**^	0.315^**^	0.459^**^	0.448^**^	0.407^**^	0.395^**^
Total P	−0.103	−0.09	0.095	0.054	0.021	−0.021
Total K	0.291^*^	0.252^*^	0.137	0.118	0.179^**^	0.142^*^
Alkali-hydrolyzable N	0.314^**^	0.287^*^	0.543^**^	0.574^**^	0.459^**^	0.461^**^
Available P	0.074	0.086	0.353^**^	0.355^**^	0.187^**^	0.139^*^
Available K	0.217	0.217	0.267^**^	0.238^**^	0.235^**^	0.175^**^
CEC	0.092	0.109	0.174^*^	0.137	0.148^*^	0.121
Clay	0.063	0.057	0.12	0.091	0.103	0.086
pH	−0.280^*^	−0.294^*^	−0.277^**^	−0.259^**^	−0.271^**^	−0.243^**^
Invertase	0.707^**^	0.688^**^	0.655^**^	0.661^**^	0.663^**^	0.673^**^
Urease	0.501^**^	0.486^**^	0.739^**^	0.699^**^	0.624^**^	0.559^**^
Phosphatase	0.749^**^	0.751^**^	0.725^**^	0.729^**^	0.712^**^	0.737^**^
Catalase	0.149	0.174	0.267^**^	0.232^**^	0.225^**^	0.219^**^
Dehydrogenase	0.811^**^	0.801^**^	0.681^**^	0.705^**^	0.690^**^	0.729^**^
GME	0.990^**^	1	0.986^**^	1	0.981^**^	1

∗ and ∗∗ represent statistical significances at the 5% and 1% levels, respectively; OM, organic matter; N, nitrogen; P, phosphorous; K, potassium; CEC, cation exchange capacity; GME, geometric mean of enzyme activities; and TEI, total enzyme activity.

**Table 5:** Multiple linear regressions between soil physicochemical properties and enzyme activity indices in surface horizon (0–20 cm) of Changwu County.

Samples	Multiple regression equation	*R* ^2^	*P*
Orchard (*n* = 75)	log_10_⁡TEI = 1.214 + 0.722 × log_10_⁡OM − 0.157 × pH − 0.131 × log_10_⁡Total P	0.491	<0.001
	log_10_⁡GME = 1.911 + 0.792 × log_10_⁡OM − 0.176 × pH − 0.136 × log_10_⁡Total P	0.483	<0.001

Cropland (*n* = 170)	log_10_⁡TEI = 1.116 + 0.409 × log_10_⁡OM − 0.197 × pH − 0.358 × log_10_⁡AN + 0.09 × log_10_⁡Available K	0.63	<0.001
	log_10_⁡GME = 1.788 + 0.399 × log_10_⁡OM − 0.216 × pH − 0.444 × log_10_⁡AN + 0.084 × log_10_⁡Available K	0.628	<0.001

Total (*n* = 245)	log_10_⁡TEI = 0.736 + 0.607 × log_10_⁡OM − 0.126 × pH + 0.38 × log_10_⁡AN − 0.061 × log_10_⁡Available P	0.53	<0.001
	log_10_⁡GME = 1.592 + 0.638 × log_10_⁡OM − 0.159 × pH + 0.256 × log_10_⁡AN − 0.046 × log_10_⁡Available P	0.528	<0.001
